# Diagnostic Value of Cytokeratin 17 during Oral Carcinogenesis: An Immunohistochemical Study

**DOI:** 10.1155/2021/4089549

**Published:** 2021-11-22

**Authors:** Sirima Sanguansin, Theerachai Kosanwat, Rachai Juengsomjit, Sopee Poomsawat

**Affiliations:** ^1^Department of Oral Biology, Faculty of Dentistry, Mahidol University, Bangkok, Thailand; ^2^Department of Oral and Maxillofacial Pathology, Faculty of Dentistry, Mahidol University, Bangkok, Thailand

## Abstract

**Background:**

Little is known about the role of cytokeratin 17 (CK17) during oral carcinogenesis. CK17 expression in oral leukoplakia (OL), the most encountered oral potentially malignant disorders and oral squamous cell carcinoma (OSCC), remains very limited. To determine the role of CK17 during oral carcinogenesis and its potential diagnostic marker in oral premalignant and malignant lesions, this study evaluated CK17 expression in OL without dysplasia, OL with dysplasia, and OSCC. CK17 expression in these tissues was compared with those of normal oral mucosa (NOM). Additionally, the relationship between CK17 expression and clinicopathologic factors of OSCC was investigated.

**Methods:**

CK17 expression was evaluated in 186 samples consisting of 12 NOM, 33 OL without dysplasia, 58 OL with dysplasia, and 83 OSCC using immunohistochemistry. The proportion of positively immunostained cells was evaluated and scored.

**Results:**

CK17 was expressed in 8.3%, 54.5%, 74.1%, and 90.4% of NOM, OL without dysplasia, OL with dysplasia, and OSCC, respectively. NOM had a significantly lower CK17 score than OL with dysplasia (*p*=0.0003) and OSCC (*p* < 0.0001). A significant association between CK17 expression and histopathologic differentiation of OSCC was found. Tumors with well differentiation had high CK17 expression compared with those of moderate and poor differentiation.

**Conclusion:**

CK17 was overexpressed in OL with dysplasia and OSCC, suggesting that CK17 plays a pivotal role in the development of premalignant lesions and OSCC. Of clinical significance, CK17 may be a good diagnostic marker for oral premalignant lesions and OSCC. Additionally, CK17 could be used as an objective tool to classify histopathologic grade in OSCC. The findings that CK17 expression is high in OSCC but low in NOM imply that CK17 may serve as a potential therapeutic target for OSCC.

## 1. Introduction

Cytokeratins (CKs) are intermediate filament proteins of the cytoskeleton of epithelial cells. CKs not only provide structural support for epithelial cells, accumulating evidence reveals that CKs also regulate cell proliferation, migration, and apoptosis. Thus, CKs participate in many physiological and pathological conditions including development, wound healing, skin diseases, and cancers [1]. The expression of CKs is extremely specific and dependent on epithelial type and stage of differentiation, making them excellent markers for cellular differentiation and cancer diagnosis [2]. Interestingly, alterations of CK expression are observed in many cancer types including oral squamous cell carcinoma (OSCC), colorectal adenocarcinoma, and Merkel cell carcinoma [2, 3].

In the CK family, cytokeratin 17 (CK17) is an interesting intermediate filament protein. Using cDNA microarray analyses of 31 CK genes, CK17 was strongly upregulated in OSCC compared to normal mucosal samples [4]. Additionally, our previous work has shown that gene expression of CK17 was in the top 20 upregulated genes among 2,724 genes [5]. The mRNA of CK17 was increased by 25 fold compared with their paired normal oral tissues. Further analysis by immunohistochemical technique revealed that CK17 was found in 100% of 39 OSCC tissue samples, whereas no staining was detected in normal oral mucosal tissue adjacent to cancerous tissue. Consistent with our study, several studies have demonstrated overexpression of CK17 both at gene and protein levels in OSCC compared with adjacent normal oral mucosa [3, 6–10]. These results indicate that CK17 is an essential cytokeratin for OSCC and CK17 may be a good candidate to diagnose OSCC. Interestingly, CK17 may also be a potential biomarker for prognosis. Elevated CK17 mRNA in peripheral blood mononuclear cells and an increased CK17 protein in oropharyngeal SCC samples were associated with decreased patient survival [11, 12]. Experiments with representative OSCC cell lines have shown that proliferation of CK17 knockdown cells was inhibited, and the cell size of these knockdown cells was significantly smaller than that of control cells [13]. Based on the above information, CK17 appears to play a crucial function in OSCC.

The role of CK17 in the early stage of oral carcinogenesis remains sparse. Oral leukoplakia (OL) is the most encountered oral potentially malignant disorder [14]. OL precedes a number of OSCC [15, 16]. A recent meta-analysis revealed that 9.7% of OL transform to OSCC [17]. Based on the English-language literature, only three groups have investigated CK17 expression in tissue samples of OL [3, 18, 19]. Kitamura and coworkers [3] revealed that strong CK17 expression was significantly found in OL with dysplasia compared to lesions without dysplasia. They discovered that CK17 was expressed in approximately 49% of OL without dysplasia and 60% of OL with dysplasia. By contrast, Okada et al. [19] found that only 10% of OL without dysplasia and 100% of OL with dysplasia were reactive to CK17. Based on 11 cases of OL and 21 cases of OSCC, Ohkura and coworkers [18] found that a remarkable CK17 expression was observed only in OSCC. Thus, the expression of CK17 in OL remains ambiguous.

To determine the role of CK17 in the early and late events of oral carcinogenesis and its potential diagnostic marker in oral premalignant lesions and OSCC, this study evaluated CK17 expression in OL without dysplasia, OL with dysplasia, and OSCC. We compared CK17 expression in these tissues with those of normal oral mucosa. To date, only one study compared CK17 expression between these four tissue types [3]. Normal oral mucosa derived from healthy persons receiving minor surgery was used in this study. We considered that this is the advantage of our study compared with several studies comparing CK17 expression between normal oral mucosa with other tissue types. Other studies used histologically normal oral mucosa adjacent to cancerous tissue as controls [6–9, 13]. This study also investigated the interrelationship between CK17 expression and clinicopathologic factors of OSCC.

## 2. Materials and Methods

### 2.1. Specimens

This work was conferred by the Ethical Board (MU-DT/PY-IRB 2018/051.0211). Cases with clinical diagnoses of OL were retrieved from departmental archives. Then, sections stained with hematoxylin and eosin of these cases were examined. Only cases with histopathologic features of hyperkeratosis and epithelial dysplasia were chosen and retrieved. Cases with hyperkeratosis were classified as OL without dysplasia, whereas cases with epithelial dysplasia were categorized as OL with dysplasia. The architectural and cytological disturbances of the epithelium were used to decide the presence of dysplasia [20]. The severity of epithelial dysplasia was graded into mild, moderate, and severe groups. Cases with histopathologic diagnosis of OSCC were retrieved as paraffin blocks. In the OSCC samples, TNM classification and histopathologic grade were determined based on the latest WHO classification [20]. Normal oral mucosal specimens were derived from healthy persons receiving minor surgery. They were fixed in 10% buffered formalin and processed under the same protocol as paraffin blocks of OL and OSCC.

### 2.2. Immunohistochemistry

Immunohistochemistry was conducted using polymer-immune complexes as described previously [21]. In brief, antigen sites were revealed by microwave pretreatment in citrate buffer solution. A mouse monoclonal anti-cytokeratin 17 antibody (clone E3, Dako, Glostrup, Denmark; dilution 1/100) was employed. Negative controls were treated by omitting the specific antibody and replacing it with phosphate buffer saline. To serve positive controls, myoepithelial cells of salivary gland tissue were used.

### 2.3. Semiquantitative Analysis of CK17 Expression

Cells showing golden brown color were considered to be positive. The proportion of positively immunostained cells was evaluated. Each case was classified into one of four scores as follows: 0 (≤5% immunoreactive cells), 1 (6–25% immunoreactive cells), 2 (26–50% immunoreactive cells), and 3 (≥51% immunoreactive cells). The semiquantitative analysis was selected for this study because it is a convenient and inexpensive technique and requires no specialized software, camera, or computer. The semiquantitative technique can also provide a comprehensive overview of the immunohistochemical staining for statistical comparison between studied groups [22]. Moreover, it has been shown that the scoring systems are reproducible and suitable to determine the predictive value of tumor biomarkers [23].

### 2.4. Statistical Analysis

Due to the nature of the ordinal scoring system, the nonparametric test was utilized in the current study. The scores of CK17 expression between the four tissue types and among different grades of epithelial dysplasia were compared using the Kruskal–Wallis test and post hoc Dunn's multiple comparison test. A linear trend of CK17 expression from normal oral mucosa to OL without dysplasia, OL with dysplasia, and OSCC was determined by chi-square test for trend. The association between CK17 expression and clinicopathologic parameters was analyzed with the Mann–Whitney test. A value of *p* < 0.05 was regarded as statistically significant. GraphPad Prism version 8.2.0 (GraphPad Software, San Diego, CA, USA) was employed for statistical analyses.

## 3. Results

### 3.1. Case Characteristics

One hundred and eighty-six samples were enrolled in the current work. They contained 12 cases of the normal oral mucosa, 33 cases of OL without dysplasia, 58 cases of OL with dysplasia, and 83 cases of OSCC. The average age of the normal mucosal subjects was 57 years with a range between 34 and 83 years. They comprised 4 males and 7 females. The age and gender of one subject in the normal oral mucosa group were unavailable. The demographic data of OL and OSCC are presented in Tables 1 and 2, respectively.

### 3.2. Morphological Pattern of CK17 in Normal Oral Mucosa, OL without Dysplasia, OL with Dysplasia, and OSCC

**Figure 1:**
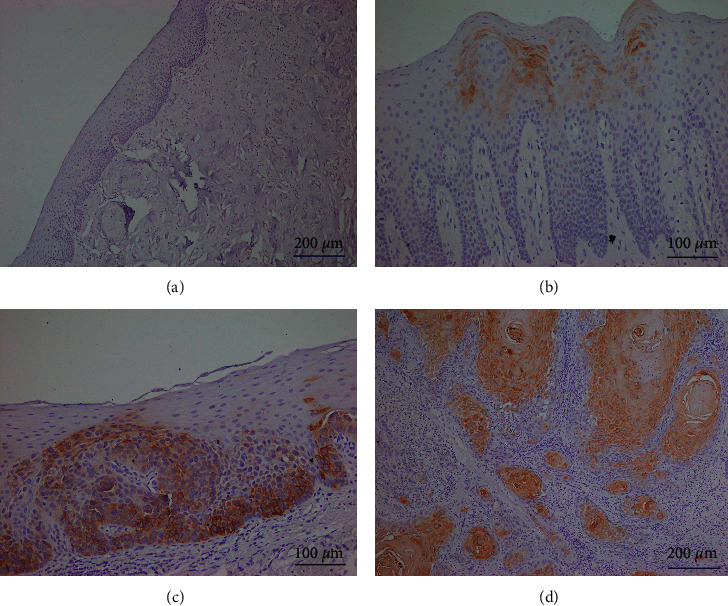
CK17 expression in representatives of the normal oral mucosa (a), oral leukoplakia (OL) without dysplasia (b), OL with dysplasia (c), and oral squamous cell carcinoma (OSCC) (d). (a) CK17 is not detected in the normal oral mucosa. In OL without dysplasia (b), CK17 is focally observed in the cytoplasm of upper prickle cells. In OL with dysplasia (c), CK17 decorates the cytoplasm of several basal cells, parabasal cells, as well as prickle cells. In well-differentiated OSCC, (d) cytoplasmic expression of CK17 is detected in numerous tumor cells at the central area of tumor islands.

**Figure 2:**
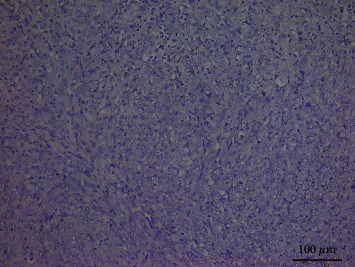
CK17 expression in a representative of poorly differentiated oral squamous cell carcinoma. Tumor cells are devoid of CK17.

CK17 was found in the cytoplasm of epithelial cells in these four tissue types. Representatives of the normal oral mucosa, OL without dysplasia, OL with dysplasia, and OSCC are displayed in Figure 1. CK17 was barely detected in the normal oral mucosa. In the majority of OL without dysplasia, the weak-to-moderate staining intensity of CK17 was occasionally found in the upper prickle cells. The localization of CK17 in the OL with dysplasia group was generally similar to those of OL without dysplasia. However, in some cases of moderate or severe dysplasia, the staining pattern clearly differed compared with that of the OL without dysplasia group. In this group, CK17 expression was found in basal and parabasal cells in addition to prickle cells. Generally, the staining intensity in OSCC was stronger than those of OL with and without dysplasia. In the well-differentiated tumors, CK17 was frequently found in the central cells of tumor islands particularly cells surrounding keratin pearls. In the moderately differentiated tumors, CK17 was also found in the central areas of tumor islands, but the staining intensity appeared to be less intense. By contrast, poorly differentiated tumors were negative to CK17 (Figure 2), or they contained only a few immunoreactive cells.

### 3.3. The Percentages of CK17 Positive Cells in Normal Oral Mucosa, OL without Dysplasia, OL with Dysplasia, and OSCC

The percentage of CK17-positive cells was graded into four scores.  Table 3 shows the CK17 score in the four tissue types. Score 3, the highest score, indicated that CK17-positive cells were expressed in more than or equal to 51%. Interestingly, of 30 cases with score 3, 29 cases were in the OL with dysplasia and OSCC groups. OSCC had a significantly higher CK17 score than normal oral mucosa (*p* < 0.0001) and OL without dysplasia (*p*=0.0012). Additionally, OL with dysplasia had a significant CK17 score than those of normal oral mucosa (*p*=0.0003). Although CK17 expression of OL with dysplasia was not significantly different from OL without dysplasia, score 3 was found in 11 cases in the OL with dysplasia group compared with only 1 case in the OL without dysplasia group. Of 11 cases of score 3 in OL with dysplasia, 8 cases were moderate or severe dysplasia. However, a significant difference in CK17 expression between different grades of epithelial dysplasia in the OL with dysplasia group was not observed (*p*=0.2859).

### 3.4. The Positive Rate of CK17 Expression in Normal Oral Mucosa, OL without Dysplasia, OL with Dysplasia, and OSCC

**Figure 3:**
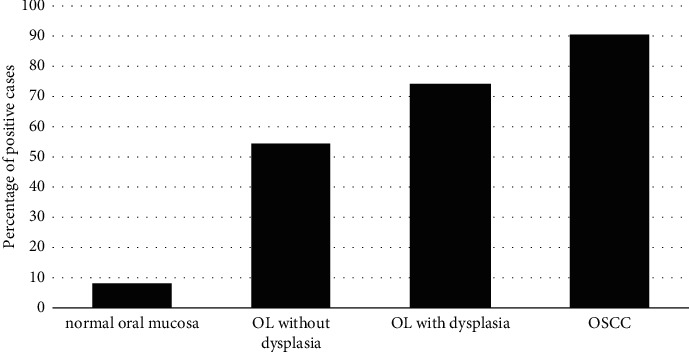
The percentage of CK17-positive cases in normal oral mucosa, oral leukoplakia (OL) without dysplasia, OL with dysplasia, and oral squamous cell carcinoma (OSCC). A linear trend of increased CK17 expression from normal oral mucosa to OL without dysplasia, OL with dysplasia, and OSCC is significantly illustrated using the chi-square test for trend (*p* < 0.0001).

Only 1 of 12 cases (8.3%) of normal oral mucosa was positive to CK17. This frequency of CK17 positive cases was lower than those of OL without dysplasia (18/33 cases; 54.5%), OL with dysplasia (43/58 cases; 74.1%), and OSCC (75/83 cases; 90.4%). An increased positive rate of CK17 expression from normal oral mucosa to OL without dysplasia, to OL with dysplasia, and then to OSCC was clearly observed as shown in Figure 3 (*p* < 0.0001).

### 3.5. The Association between CK17 Expression and Clinicopathologic Parameters of Patients with OSCC

The high positive rate of CK17 expression in OSCC suggests that CK17 may have clinical implications. Therefore, we further investigated the relationship between CK17 expression and clinicopathologic variables of OSCC. As shown in Table 4, CK17 expression was significantly correlated with histopathologic differentiation of OSCC (*p*=0.0143). High CK17 expression was frequently found in tumors with well differentiation compared with tumors with moderate and poor differentiation. No significant association was found between CK17 and other clinicopathologic parameters including age, sex, location, clinical stage, tumor size, and regional lymph node involvement.

## 4. Discussion

This study demonstrated that CK17 was expressed in 8.3%, 54.5%, 74.1%, and 90.4% of the normal oral mucosa, OL without dysplasia, OL with dysplasia, and OSCC, respectively. A significant trend of increased CK17 expression from normal oral mucosa to OL without dysplasia, OL with dysplasia, and OSCC was observed. Normal oral mucosa had a significantly lower CK17 score than OL with dysplasia and OSCC. CK17 expression significantly correlated with histopathologic grade in OSCC. Tumors with well differentiation had high CK17 expression compared with tumors of moderate and poor differentiation.

CK17 expression in normal oral mucosa derived from healthy tissue remains little known. Almost all studies of CK17 expression used histologically normal oral mucosa adjacent to cancerous areas [13, 24, 25], except for Kitamura's study [3]. Consistent with Kitamura et al. [3], our result showed that CK17 was barely detected in the normal oral mucosa. Several studies using histologically normal oral mucosa also reported the negative staining of CK17 in normal oral mucosa [13, 24, 25]. Therefore, it is likely that CK17 does not have any function in the normal oral mucosa.

Approximately 55% of OL without dysplasia was immunoreactive to CK17. This finding is in concordance with a related work reporting a positive rate of 49% in hyperplastic leukoplakia [3] but is inconsistent with another study showing that only 10% of OL without dysplasia was positive to CK17 [19]. However, the latter study had only 10 cases of OL without dysplasia. This sample size was relatively small for statistical analysis. In the current work, CK17 expression was predominantly found in the prickle cell layer in this tissue type. This staining pattern is similar to a previous study [26]. Based on this evidence, it appears that the expression of CK17 is induced in the hyperplastic epithelium. These results are supported by the evidence that CK17 expression is upregulated in epithelial cells in the hyperproliferative status including wound healing [27] and viral infections [28]. Although an increased expression of CK17 in OL without dysplasia was observed, the expression did not significantly differ from normal oral mucosa. Therefore, CK17 is not a helpful marker in diagnosing OL with histopathological features of hyperkeratosis.

Approximately 74% of OL with dysplasia showed positive immunoreactivity to CK17. This figure is comparable to previous studies showing a positive rate of 60 to 100% in the OL with dysplasia group [3, 19]. Among these studies, our study contained the largest sample size of OL with dysplasia. The overexpression of CK17 in OL with dysplasia compared with normal oral mucosa from our study is consistent with previous studies [3, 19], indicating that CK17 is involved in the dysplastic process in OL. Additionally, CK17 may be the cause of the dysplastic process. It has been postulated that cell migration induced by CK17 might lead to architectural disturbances in the dysplastic process [8]. Interestingly, foci of epithelial dysplasia adjacent to cancerous tissue also showed increased expression of mRNA and protein of CK17 compared with histologically normal oral mucosa [13, 24]. Additionally, it has been shown that epithelial dysplasia adjacent to OSCC has the potential to develop cancer, and this tissue type shares some properties with the early stage of OSCC [29]. Taken all together, CK17 is a useful biomarker to diagnose OL with dysplasia. Importantly, CK17 may be a potential marker to early detect OSCC. However, the limitation of this study was that CK17 could not be used as a marker to predict which OL will progress to OSCC. Therefore, prospective studies using large sample sizes would be very useful to evaluate the role of CK17 as a predictive marker for malignant transformation.

A significant difference in CK17 expression among the three grades of epithelial dysplasia in the OL group was not observed in this study. This result is in line with two studies comparing CK17 expression in various grades of epithelial dysplasia in OL [3, 18]. Ohkura et al. [18] reported that the level of mRNA of CK17 in severe dysplasia was significantly higher than those of hyperplasia and mild-to-moderate epithelial dysplasia, but this difference was undetected at the protein level. Possibly, the histomorphological changes do not correlate with the phenotypical alterations.

The staining pattern of CK17 in OL with dysplasia also drew our attention. We observed that CK17 was located in basal and suprabasal cells of some moderate and severe epithelial dysplasia, whereas it was confined to the prickle cell layer in mild epithelial dysplasia. Previous works have also demonstrated that the basal staining of CK17 was found in high-grade epithelial dysplasia or carcinoma in situ but not in low-grade lesions [13, 26]. Because this staining pattern was unobserved in any cases of OL without epithelial dysplasia and OL with mild epithelial dysplasia, this characteristic may be useful in separating high-grade epithelial dysplasia from low-grade lesions. This staining pattern may also solve the problem of subjectivity in histopathological grading of epithelial dysplasia.

CK17 expression in OL with and without dysplasia in our study is somewhat equivalent to CK17 expression observed in cutaneous warts [28]. CK17 expression was found in the suprabasal layer in hyperproliferative lesions, whereas its expression was discovered in the basal plus suprabasal cells in dysplastic warts. Moreover, dysplastic warts with basal CK17 expression subsequently developed to SCC. Based on our results and observations in cutaneous models, we, therefore, suggest that OL with a combination of strong CK17 expression and basal staining of CK17 may have a high potential for neoplastic change.

Although several studies have demonstrated the aberrant expression of CK17 in OSCC in comparison with the normal oral mucosa, almost all of these studies used histologically normal oral tissue adjacent to cancerous areas. According to the field cancerization concept, the histologically normal oral mucosa adjacent to malignant tissue may not be a good candidate as compared with the normal oral mucosa of healthy tissue. At present, only one study used normal oral mucosa from healthy volunteers [3]. Thus, CK17 expression in OSCC in relation to the normal oral mucosa of healthy tissue remains little known. Similar to a previous study [3], our study found elevated CK17 expression in OSCC compared to the normal oral mucosa. These observations imply that CK17 has a key function in OSCC. The high positive rate of CK17 in OSCC found in our study (90.4%) as well as several studies (79–100%) strongly supports that CK17 plays a pivotal role in OSCC [3, 5, 6, 9, 24]. Additionally, CK17 may be an essential protein in OSCC. Of clinical importance is that CK17 has a high potential to be a biomarker to diagnose OSCC. Moreover, the high positive rate of CK17 expression in OSCC and the low positive rate in normal oral mucosa observed in this study as well as other studies suggest that CK17 may be an excellent therapeutic target for OSCC.

An increased expression of CK17 with the severity of lesions from normal mucosa to OL without dysplasia, OL with dysplasia, and OSCC was significantly demonstrated in the current work. A similar trend of results was also described by Kitamura and associates [3], but the statistical analysis was not performed in their study. These findings imply that CK17 may participate in the tumorigenesis process of OSCC. Results from a study using an animal model support this notion [30]. After carcinogen exposure, mice that developed SCC showed strong CK17 expression, while the control mice were negative to CK17. It has been postulated that CK17 participates in tumorigenesis via the regulation of cytoskeletal remodeling during cell growth and cell migration [30]. Analysis of an OSCC cell line has shown that CK17 promotes cell propagation and motility by the activation of the AKT/mammalian target of rapamycin (mTOR) pathway. Moreover, CK17 elevated glucose uptake and the expression of solute carrier family 2 member 1/Glut1. Tumors from CK17-knockout cells had a lower Ki-67 index and were significantly smaller than those of control cells. These findings imply that tumor growth is promoted by glucose uptake induced by CK17 via the AKT/mTOR pathway [7]. Another study also found that CK17 stimulated cell proliferation and the cell size of CK17-knockdown cells was significantly smaller than that of control cells [13]. Additionally, CK17 has been shown to enhance migratory and invasive capacities of oral carcinoma cells by modulation of the epithelial-mesenchymal transition [30]. The role of CK17 in tumorigenesis in OSCC is advocated by studies in the SCC of the cervix. CK17 expression significantly increased from normal cervical squamous mucosa to low-grade squamous intraepithelial lesions, to high-grade squamous intraepithelial lesions, and to SCC [31]. Additionally, CK17-negative premalignant lesions did not transform to SCC, whereas a high proportion of CK17-positive patients developed SCC [32].

It appears that CK17 does not act alone during oral carcinogenesis; an interplay between CK17 with several factors known to play a role in carcinogenesis is likely to be another mechanism to promote oral carcinogenesis. The expression of CK17 is regulated by glioma-associated oncogene-2, known to act as an oncogene in OSCC [33]. Additionally, CK17 controls the expression of the autoimmune regulator, a transcriptional regulator responsible for CK17-induced inflammation in tumorigenesis [34]. CK17 functions as an oncoprotein through binding with the cell cycle inhibitor p27^KIP1^. In human cancers, the level of p27^KIP1^ is frequently interfered by MAPK, SRC, and receptor tyrosine kinase tracks [35].

Apart from the function of CK17 in promoting tumorigenesis in the early and late events of oral carcinogenesis, CK17 appears to participate in the differentiation process in OSCC. Tumors with well differentiation had high CK17 expression compared with those of moderate and poor differentiation. Our results are in accordance with two studies [3, 36]. Additionally, we and several other groups [3, 9, 13, 36] observed that CK17 was detected in the central cells of tumor islands particularly tumor cells surrounding keratin pearls. These tumor cells were less frequently observed in moderately and poorly differentiated tumors. This observation explains why tumors with well differentiation showed high CK17 expression compared with tumors with higher grades. The findings that CK17 mRNA in HSC-2, a cell line obtained from OSCC with well differentiation, was significantly higher than those in HSC-3 and SAS, cell lines obtained from OSCC with poor differentiation support our notion [3]. Based on our results together with those of previous studies, CK17 expression could possibly be used as an objective tool to classify histopathologic grades for OSCC.

## 5. Conclusions

CK17 was overexpressed in OL with dysplasia and OSCC. A gradual increase of CK17 expression from normal oral mucosa to OL without dysplasia, OL with dysplasia, and OSCC was also demonstrated. These results suggest that CK17 plays a pivotal function in the early and late stages of oral carcinogenesis and is involved in the development of premalignant lesions and malignant transformation. CK17 may be used as a diagnostic marker for oral premalignant lesions and OSCC. Compared with moderately or poorly differentiated tumors, well-differentiated OSCC had high CK17 expression, suggesting that CK17 could be used as an objective tool to classify histopathologic grade in OSCC. Future studies using large sample sizes will provide translational implications before using CK17 as a diagnostic marker and as a therapeutic target.

## Figures and Tables

**Table 1:** Demographic data of OL without dysplasia and OL with dysplasia.

Characteristic	Category	OL without dysplasia	OL with dysplasia
		Cases (%)	Cases (%)
Sex	Male	21 (63.6)	28 (48.3)
	Female	12 (36.4)	30 (51.7)

Age	Average (range)	55.6 (28–79)	60.7^a^ (33–87)

Degree of epithelial dysplasia	Mild	—	27 (46.6)
	Moderate	—	15 (25.9)
	Severe	—	16 (27.6)

Localization of the lesion	Palate	6 (18.2)	20 (34.5)
	Tongue	2 (6.1)	17 (29.3)
	Gingiva	6 (18.2)	7 (12.1)
	Buccal mucosa	6 (18.2)	7 (12.1)
	Alveolar mucosa	9 (27.3)	5 (8.6)
	Lip	0 (0)	2 (3.4)
	Retromolar	4 (12.1)	0 (0)

^a^The ages of the three cases are unavailable.

**Table 2:** Demographic data of OSCC.

Characteristic	Category	Cases (%)
Sex	Male	50 (60.2)
	Female	33 (39.8)

Age	Average (range)	55.7 (19–92)

Histopathologic differentiation	Well	44 (53.0)
	Moderate	37 (44.6)
	Poor	2 (2.4)

Localization of the lesion	Tongue	42 (50.6)
	Buccal mucosa	11 (13.3)
	Palate	11 (13.3)
	Gingiva	7 (8.4)
	Floor of the mouth	4 (4.8)
	Lip	3 (3.6)
	Retromolar	2 (2.4)
	Socket	2 (2.4)
	Alveolar mucosa	1 (1.2)

**Table 3:** Score of CK17 expression among four tissue types.

Tissue type	Number of cases	Score			
		0	1	2	3
Normal oral mucosa^*∗*^,^*∗∗∗*^	12	11	1	0	0
OL without dysplasia^*∗∗*^	33	15	9	8	1
OL with dysplasia	58	15	17	15	11
OSCC	83	8	32	25	18

^*∗*^ *p* < 0.0001 and ^*∗∗*^*p*=0.0012, statistically significant difference compared with OSCC. ^*∗∗∗*^*p*=0.0003, statistically significant difference compared with OL with dysplasia. OL = oral leukoplakia; OSCC = oral squamous cell carcinoma.

**Table 4:** Association between CK17 expression and clinicopathologic parameters of patients with OSCC.

Clinicopathologic parameters	No. of patients (%)	0	1	Score	2	3	*p* value
*Age* ^*a*^							
≤60	50 (60.2)	5	19		14	12	0.7036
>60	33 (39.8)	4	13		9	7	

*Sex* ^*a*^							
Male	50 (60.2)	5	21		15	9	0.3456
Female	33 (39.8)	3	11		10	9	

*Localization* ^*a*^							
Tongue	42 (50.6)	2	17		13	10	0.3641
Nontongue	41 (49.4)	6	15		12	8	

*Clinical stage* ^*b*^							
Stages 1 + 2	24 (35.3)	3	10		6	5	0.4789
Stages 3 + 4	44 (64.7)	4	15		15	10	

*T classification* ^*b*^							
T1 + T2	46 (67.6)	5	19		13	9	0.2851
T3 + T4	22 (32.4)	2	6		8	6	

*Regional lymph node metastasis* ^*b*^							
Negative	30 (44.1)	4	12		7	7	0.5142
Positive	38 (55.9)	3	13		14	8	

*Histopathologic grade* ^*a*^							
Well	44 (53.0)	3	12		17	12	0.0143^*∗*^
Moderate + Poor	39 (47.0)	5	20		8	6	

^*∗*^Statistically significant. ^a^83 cases. ^b^68 cases.

## Data Availability

The statistical data used to support the findings of this study are included within the article.
